# Functional Remineralization of Dentin Lesions Using Polymer-Induced Liquid-Precursor Process

**DOI:** 10.1371/journal.pone.0038852

**Published:** 2012-06-13

**Authors:** Anora K. Burwell, Taili Thula-Mata, Laurie B. Gower, Stefan Habeliz, Michael Kurylo, Sunita P. Ho, Yung-Ching Chien, Jing Cheng, Nancy F. Cheng, Stuart A. Gansky, Sally J. Marshall, Grayson W. Marshall

**Affiliations:** 1 Department of Preventive and Restorative Dental Sciences, University of California San Francisco, San Francisco, California, United States of America; 2 Materials Science and Engineering Department, University of Florida, Gainesville, Florida, United States of America; University of Toronto, Canada

## Abstract

It was hypothesized that applying the polymer-induced liquid-precursor (PILP) system to artificial lesions would result in time-dependent functional remineralization of carious dentin lesions that restores the mechanical properties of demineralized dentin matrix. 140 µm deep artificial caries lesions were remineralized via the PILP process for 7–28 days at 37°C to determine temporal remineralization characteristics. Poly-L-aspartic acid (27 KDa) was used as the polymeric process-directing agent and was added to the remineralization solution at a calcium-to-phosphate ratio of 2.14 (mol/mol). Nanomechanical properties of hydrated artificial lesions had a low reduced elastic modulus (E_R_ = 0.2 GPa) region extending about 70 μm into the lesion, with a sloped region to about 140 μm where values reached normal dentin (18–20 GPa). After 7 days specimens recovered mechanical properties in the sloped region by 51% compared to the artificial lesion. Between 7–14 days, recovery of the outer portion of the lesion continued to a level of about 10 GPa with 74% improvement. 28 days of PILP mineralization resulted in 91% improvement of E_R_ compared to the artificial lesion. These differences were statistically significant as determined from change-point diagrams. Mineral profiles determined by micro x-ray computed tomography were shallower than those determined by nanoindentation, and showed similar changes over time, but full mineral recovery occurred after 14 days in both the outer and sloped portions of the lesion. Scanning electron microscopy and energy dispersive x-ray analysis showed similar morphologies that were distinct from normal dentin with a clear line of demarcation between the outer and sloped portions of the lesion. Transmission electron microscopy and selected area electron diffraction showed that the starting lesions contained some residual mineral in the outer portions, which exhibited poor crystallinity. During remineralization, intrafibrillar mineral increased and crystallinity improved with intrafibrillar mineral exhibiting the orientation found in normal dentin or bone.

## Introduction

Replacing mineral within type I collagen is critical to establishing the normal mechanical properties of calcified tissues such as dentin that forms the bulk of the tooth [1], [2]. Much of the work in this area has focused on the use of calcium phosphate solutions to form apatite by classical nucleation and growth methods [3], [4], including recent work that has shown promise of restoration of normal mechanical properties in hydrated tissue, a process termed “functional remineralization" [5].

Gower and colleagues have suggested a different paradigm based on formation of a polymer-induced liquid- precursor (PILP) system. Although several other groups have suggested that amorphous inorganic precursors may be a critical stage in biomineralization, Gower's group pioneered the concept that formation of liquid nanoprecursors encapsulated by polyanionic polymers may be a fundamental step in many forms of biomineralization [6], [7], [8]. These studies have shown significant success in mineralizing a variety of organic matrices with both calcium carbonate and calcium phosphates including collagen matrices mineralized by formation of hydroxyapatite [9].

The PILP process entails adding micromolar quantities of acidic polypeptides to the remineralization solution. The anionic polymer sequesters calcium ions, which then builds up a charge to sequester counter ions (phosphate or carbonate, depending on the system), which induces liquid-liquid phase separation in the crystallizing medium [8], [10], [11]. This study exploits the concept of “biologically induced mineralization" using PILP mechanism for remineralization of dental carious lesions.

Dental caries is the most prevalent chronic infectious disease in humans (http://www.nidcr.nih.gov/DataStatistics/FindDataByTopic/DentalCaries/) and results in the destruction of the calcified tissues of the tooth (enamel, dentin and cementum). Although early intervention allows remineralization of enamel, if the disease progresses to dentin, current practice requires surgical removal of carious dentin and placement of a dental restoration. Much current work is focused on the complex task of remineralization of dentin, which, if successful, would minimize surgical intervention and preserve much of the tooth tissue. Few studies have attempted to define the essential metrics for load bearing integrity of calcified tissues. One such metric is based on a fundamental mechanical property, namely indentation elastic modulus as measured in hydrated tissues [1], [2], and in this work recovery of this property was determined after applying the PILP process to artificial carious lesions. Thus functional remineralization is the result of a process that yields recovery of physical and chemical properties otherwise lost due to disease. The PILP delivery process is highly effective at mineralizing type I collagen scaffolds with apatite including those that do not contain remnant mineral, such as reconstituted collagen sponges [6], [9], [12], [13] and turkey and bovine tendon [9].

Dentin is a calcified tissue, similar in composition to bone, and is composed mainly of collagen fibrils reinforced with apatite with additional apatite between the collagen fibrils (extrafibrillar mineral estimated at 70–75% of the total mineral [1]). The mineral formed within the collagen fibrils has the same [001] crystallographic orientation as found in bone. Recent work suggests that collagen can direct the crystallization process once it is infiltrated with the amorphous precursor [14]. Similar approaches have used Portland cement/phosphate-containing fluid system in the presence of polyacrylic acid and polyvinylphosphonic (PVPA) at pH of 9.25 to produce intrafibrillar and extrafibrillar remineralization of phosphoric acid-etched dentin [15]. These studies have been aimed mainly at remineralization of the collagen in bonded hybrid layers that are common in dentin bonding procedures and that are susceptible to degradation due to hydrolytic instability and/or enzymatic activity. In such studies it is proposed that the polyacrylic acid stabilizes amorphous calcium phosphates and that PVPA acts as a collagen-binding matrix protein to mimic natural non-collagenous proteins that control biomineralization. Extrafibrillar and intrafibrillar remineralization of phosphoric acid-etched human dentin have been demonstrated using this technique [16], [17]. Recently, mineralization of reconstituted collagen fibrils on TEM grids using this method has been shown as well [18].

Functional remineralization of lesions should restore the elastic modulus values and produce a mineralized matrix that contains intrafibrillar mineral oriented as found in normal dentin and bone. In this work it was hypothesized that applying the PILP system to artificial lesions would result in time-dependent functional remineralization of carious dentin lesions. To test this hypothesis, 140 μm deep artificial caries lesions were prepared and remineralized for periods of 7, 14, and 28 days. The hydrated specimens were evaluated by nanoindentation [2], [19], [20] and the extent of mineralization was determined using MicroXCT™.

## Materials and Methods

### 2.1 Specimen Preparation – Artificial caries lesion model

**Figure 1:**
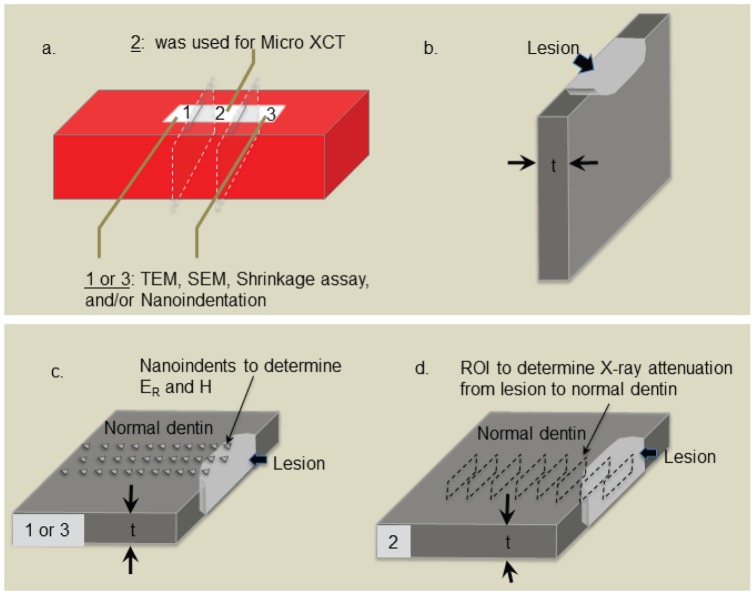
Schematic diagram showing specimen preparation and analyses. a) dentin block covered with nail varnish with exposed window for lesion formation showing subsequent sections 1–3 used for nanoindentation, MicroXCT™ and microscopy. b) Schematic of lesion section such as that labeled 1 in a). c) nanoindentations at 4 µm intervals made through lesion into normal dentin. d) regions of interest used from a thinned region of lesion marked 2 in a) that was used to determine X-ray attenuation from MicroXCT™ tomograms at intervals of ∼ 785 nm (not drawn to scale).

Permanent, fully-formed human third molars were obtained from the UCSF dental hard tissue specimen core according to protocols approved by the UCSF Committee on Human Research. After extraction the teeth were sterilized with gamma radiation and stored intact in de-ionized water and thymol at 4°C [21]. Dentin blocks measuring 4.5 mm in length and width and 2 mm in thickness were cut from the mid-coronal region of the selected teeth perpendicular to the tubule direction. The specimen surfaces to be exposed to artificial caries formation and remineralization were ground with SiC abrasive papers from 320 to 1200 grit, and then polished with aqueous diamond suspensions (Buehler, Lake Bluff, IL) of 6.0, 3.0, 1.0, and 0.25 µm particle sizes. Each specimen surface was covered with nail varnish (Revlon Nail Enamel #270, New York, NY) to prevent demineralization except for a window measuring 2.5×2.5 mm (Fig. 1a). Artificial carious lesions approximately 140 µm deep were induced by exposing the surface to a demineralizing solution consisting of 0.05 M acetate buffer containing 2.2 mM calcium phosphate [22] and adjusted to pH 5.0 for 66 hours, a demineralization treatment determined by prior kinetics studies (Fig. 1b). After artificial caries lesions were produced, the specimens (n = 4/group) were remineralized for periods of 7,14 or 28 days using the PILP mineralization process and were subsequently studied by AFM-based nanoindentation and micro x-ray computed tomography (MicroXCT™, Xradia Inc., Pleasanton, CA) to evaluate the mechanical properties recovery and mineral content. These data were compared to controls consisting of the un-remineralized lesion group (n = 4) and normal untreated dentin from the area protected by nail varnish during demineralization, (n = 4).

### 2.2 Remineralization Experiments

The PILP remineralization solutions were prepared following a slightly modified procedure [9]. Poly-L-aspartic acid (Pasp) with a molecular weight of 27 KDa was used as the polymeric process-directing agent. Calcium chloride dihydrate was dissolved in Tris buffered saline (TBS) at a 9mM concentration and Pasp was added to a final concentration of 100 μg/mL. An equal volume of dipotassium phosphate solution was added to the calcium-polymer mix, resulting in a calcium-to-phosphate ratio of 2.14 (mol/mol). Specimens were incubated at 37°C under continuous stirring for 7, 14, or 28 days. pH of the PILP system was 7.4 at the start of mineralization experiments and at the end of experiments was typically 7.1–7.2.

### 2.3 AFM-based nanoindentation to determine mechanical recovery of tissues

**Figure 2:**
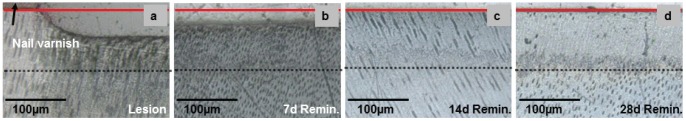
Shrinkage decreased with increased remineralization. Optical images of dried cross sections exposing the lesion depths. a) demineralized lesion with nail varnish shown protecting the unexposed surface; b) shrinkage was greatly decreased at 7days and was almost undetectable at c) 14 days; or d) 28 days. Solid red line  =  original surface location, black dotted line  =  lesion depth.

The blocks containing the lesions (Fig 1a) were embedded in room temperature curing epoxy (Epoxicure, Buehler, Ltd, Lake Bluff, IL, 60044 Resin Lot#681-501252, Hardener Lot#679-501230. The blocks were cut perpendicular to the surface to expose the lesion structure from the most demineralized outer portion through the lesion and into sound dentin using a low speed water-cooled diamond saw (Buehler; Lake Bluff, IL 60044). The blocks were cut into three separate sections. The central section (Fig 1a, 2) was cut thin and used for imaging mineral variations by MicroXCT™ as described below, while the remaining sections (Fig. 1a 1,3) were used for nanoindentation or other experiments.

The cross sections (minimum thickness of 400 µm) were fixed to metal AFM discs using a very thin layer of cyanoacrylate (MDS Adhesive QX-4 (MDS Products, Inc., Laguna Hills, CA, 92653, batch D18A) and prepared for nanoindentation by polishing as described above in Section 2.1. Prior to the start of nanoindentation analysis, the cross-sections were hydrated in de-ionized water for one hour. Wet nanoindentation (n = 4 specimens/ group) was performed in a liquid cell filled with de-ionized water using an AFM (Nanoscope III Veeco Instruments, Santa Barbara, CA) to which a load-displacement transducer (Triboscope, Hysitron Incorporated, Minneapolis, MN) was attached. A sharp diamond Berkovich indenter with a conventional radius of curvature less than 100 nm (Triboscope, Hysitron Incorporated, Minneapolis, MN) was fitted to the transducer. Fused silica was used to calibrate the transducer under dry and wet conditions. Site-specific measurements of reduced elastic modulus (E_R_) and hardness (H) were made using a controlled force of 300 µN with a 3-second trapezoidal loading profile (load, hold, and unload) as is our standard practice [1], [20], [23], [24]. Indentations were made at intervals of 4 μm starting from most demineralized outer surface of the lesion and proceeding inwards through the depth of the lesion and into sound dentin, covering a total distance of 180 µm (Fig. 1c). Similar lines of indents were placed in the normal, untreated portions of the dentin specimen, which had been protected by nail varnish during the demineralization and remineralization processes. Thus each specimen contained two lines of indents showing the variation in reduced elastic modulus (and hardness) through the lesion and one line of indents along the unaffected dentin away from the lesion.

**Figure 3:**
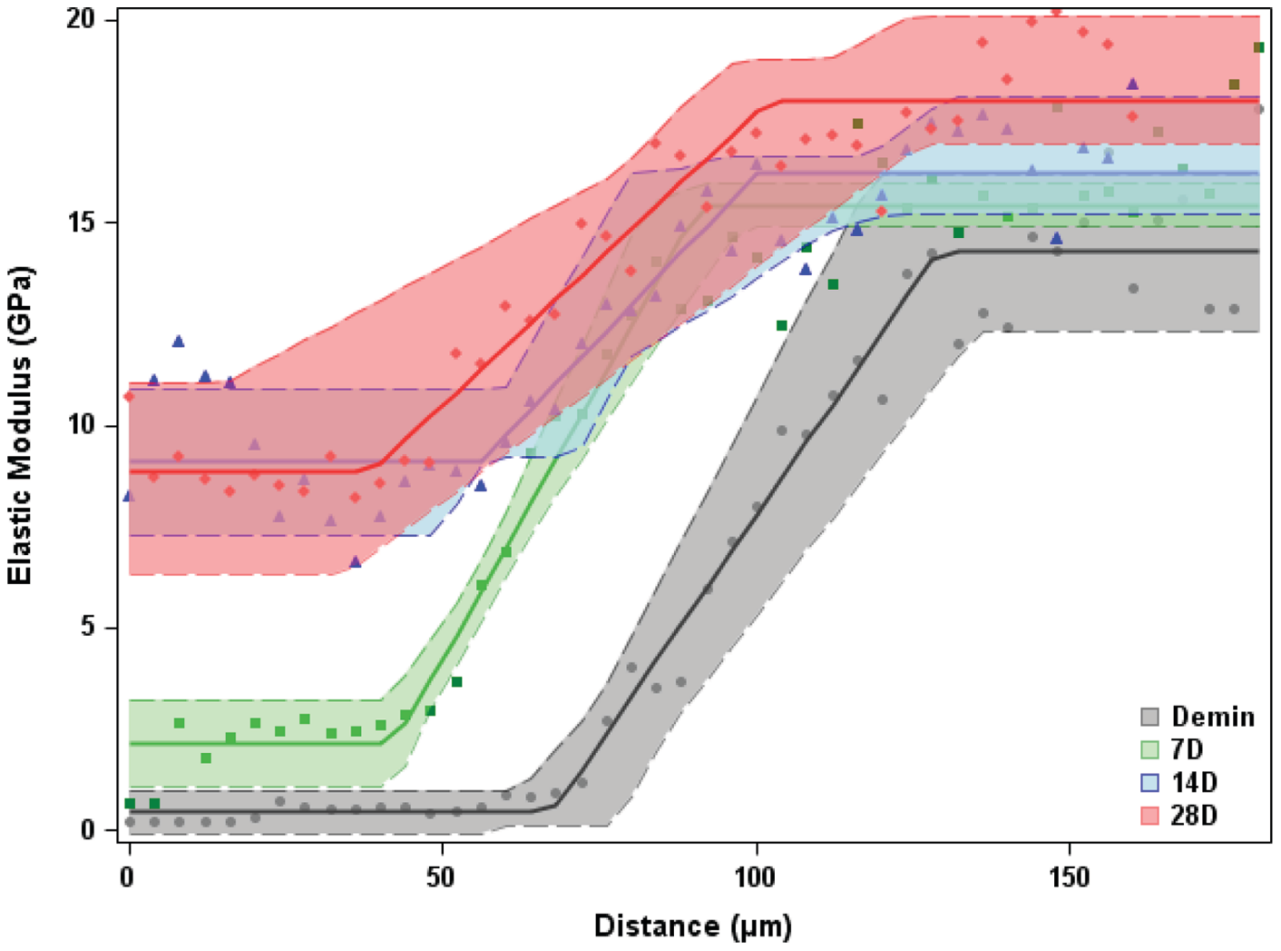
Reduced elastic modulus values increased with remineralization time as shown by statistical change-point diagrams with 95% CLs. Artificial lesion profiles (grey) had a low modulus region that extended nearly 80 µm, followed by a sloped region that extended to the depth of the lesion at 140–150 µm, Seven day PILP remineralization (green) showed significantly increased properties with normal dentin values starting at about 100 µm from the surface. These trends continued with time at 14 days (blue) and 28 days (red) of remineralization. Little additional change was observed between the 14 and 28 day treatments that had restored normal properties from about 85 µm inward. In the outer portion modulus values were restored to about half of normal levels. Average control values (open circles, squares, triangles, and diamonds) are shown without error bars for clarity.

**Figure 4:**
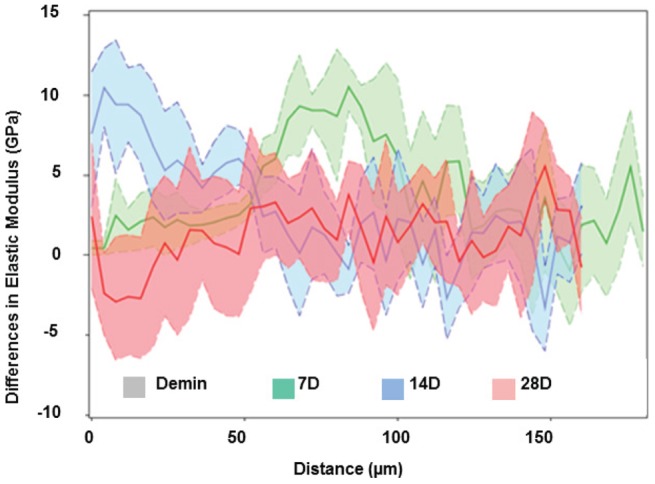
Change-point plots for the data in **Figure 3** **.** Incremental changes in E_R_ between remineralization intervals reveals that within 7days values increased predominantly in the sloped zone of the lesion (∼70–140 µm), while the 7–14 day period introduced mineral at the surface and final period between 14 and 28 days contributed only minimally to the total regain of stiffness across the lesion.

Following mechanical characterization, the cross-sections were imaged using reflective light microscopy to determine how much (if any) the surface of the dentin had shrunk during the epoxy-embedding procedure. Embedding was necessary to cut the dentin blocks perpendicular to the treated surfaces. The cross-sections were imaged at 10X with an Olympus BX 51 microscope (Olympus America Inc., San Diego, CA) and the distance between the original, intact dentin surface on the nail varnish protected and the treated dentin was measured using ImagePro Plus software (Media Cybernetics Inc., Silver Spring, MD). Two images were captured from each sample and 5 measurements were gathered from each image, for a total of 10 measurements per sample. Figure 2 contains examples of these light microscopy images, where the solid line represents the original, intact dentin surface. The individual shrinkage values for each specimen were used to provide a corrected location for the starting surface position. The unremineralized artificial lesions underwent the most shrinkage (Fig. 2a) as indicated by the difference between the solid line marking the control area and the black dots representing the interface between the lesion and normal dentin. Shrinkage decreased markedly with remineralization after 7 days (Fig. 2b) and was barely detectable at 14 or 28 days (Fig. 2c, 2d). This suggests that shrinkage is a reasonable indicator of mineral incorporation within the lesion. With mineralization, the water becomes displaced by mineral crystals so that less volumetric shrinkage is possible. In addition it shows that the apparent depth of the lesion would be incorrect if the measurements were made on dry tissue. The nanoindentation profiles presented in Figs. 3 and 4 were corrected for each specimen's shrinkage. Since the depth of shrinkage was different for each specimen, and varied with length of PILP treatment, this procedure ensured that all mechanical data were reported with the same starting point and on the same scale. There was no measurable shrinkage in hydrated specimens imaged by MicroXCT™.

To analyze the changes in mechanical properties as influenced by each treatment, the reduced elastic modulus (E_R_) measurement was plotted against distance from the specimen surface (in 4µm intervals), and the area under the curve (AUC) was calculated (GPa-µm). Percent improvement of E_R_ was calculated by comparing the AUC of the PILP-treatment groups (7, 14, and 28 days) to the AUC of the untreated artificial lesion group Hardness and elastic modulus (and their differences from control) were also statistically analyzed using mixed effects change- point models with random sample effects and two change points (initial lesion-lesion gradient and lesion gradient-normal dentin) [25]; 95% confidence limits (CLs) were estimated with a non-parametric bootstrap procedure with 1000 resamples. (Parametric bootstrap was used as a sensitivity analysis. Non-overlapping CLs indicate significant differences between the time point groups.

### 2.4 Micro x-ray computed tomography (MicroXCT™) to determine extent of mineralization

The thin cross-sections from the centers of the dentin specimens (section 2 in Fig. 1a) (n = 3 for each experimental condition) were prepared for analysis using MicroXCT™ (Xradia, Inc., Pleasanton, CA). Ground sections were prepared with SiC abrasive papers from 600 to 2400 grit to a thickness of ∼100 µm. Each ground section was analyzed with polarized light microscopy before and after the razor blade excision to confirm the lesion depth and specimen integrity. The specimens were thoroughly rinsed and placed into a micropipette tip filled with de-ionized water (Fisher Scientific, Pittsburgh, PA) to simulate physiological conditions and imaged [20], [26] (see supplementary data Fig. S1).

MicroXCT™ was carried out with a tungsten anode x-ray tube at 60 KVp, 133µA using a 10X objective and 150 µm quartz filter. The tomography data for each specimen and calibration standard was collected at an exposure of 25 seconds over 1900 scans between the angles of −95° to 95° with an average spatial resolution of ∼785 nm. Tomographs were generated and the linear attenuation from virtual slices was converted to standard Hounsfield Units (HU; air + pipette  = 0, water + pipette  = 1000). Each de- and remineralization specimen (n = 3 per condition) was scanned.

The x-ray attenuation data of each specimen as a function of depth from the exposed surface was performed by comparing the CT-scaled data using Xradia 3D viewer (Xradia, Inc. Pleasanton, CA). Equivalent regions of interest (ROI) for all specimens were used and the average HU values through the lesion of all specimens were compared. The ROI size, placement, and shape were centralized throughout the volume, paying special attention to avoid beam hardening artifacts.

Using the same approach as described for the mechanical data, the AUC (HU-µm) from the HU vs. distance curve for each time point (7, 14, and 28 days) was calculated. Percent mineralization was calculated by comparing the AUC of the PILP-treatment groups (7, 14, and 28 days) to the AUC of the untreated artificial lesion group. Data were also statistically analyzed using one-way ANOVA and Sidak-Holm statistical methods (p<0.05) [27].

### 2.5 Microscopy

Selected specimens (n = 2 per group) from the artificial lesion and remineralization groups were evaluated by scanning electron microscopy coupled with energy dispersive x-ray spectrometry (SEM/EDS) to characterize structural variations and identify important differences in calcium and phosphate content. Specimens were coated with a 10–20 nm thick Au thin film using a sputter coater (Denton Vacuum Inc., Model # Desk II, Moorestown, NJ) and imaged using a Hitachi S-4300 field-emission gun scanning electron microscope (Hitachi High Technologies America, Pleasanton, CA) at an accelerating voltage of 10 kV and using working distances of <12 mm. The EDS analyses were performed using a Noran System 6 EDS x-ray detector (Thermoscientific, Waltham, MA). Transmission electron microscopy (TEM) of 70 nm thick sections prepared from 2 specimens per group were also carried out to determine the lesion and remineralized specimen structures and selected area electron diffraction (SAED) was used to identify the nature and crystallinity of the mineral formed by remineralization treatments. Sections from either artificial lesion or remineralization groups were embedded after ethyl alcohol and then acetone dehydration in Spurr's resin (Ted Pella, Redding, CA). Selected regions were trimmed, and ultrathin sections (70 nm) were cut in occlusal and sagittal planes with a diamond knife on an ultramicrotome (Reichert-Jung Ultracut E, Leica, Wetzlar, Germany). Tissue sections were placed on Formvar™ copper grids and examined in a JEOL JEM 1400 TEM (JEOL Ltd, Tokyo, Japan) at an accelerating voltage of 120 kV. Images were recorded using a CCD camera (Gatan Inc., Pleasanton, CA).

## Results

The treatment of the dentin surface with the artificial caries acetate buffer yielded reproducible demineralized lesions of approximately 140 μm in depth, based on changes in nanomechanical properties. The exposed surfaces were prepared to be perpendicular to the dentin tubule direction. This resulted in an exposed lesion surface with widened tubule lumens as the peritubular dentin was removed preferentially, leaving a partially demineralized dentin matrix. Subsequent analyses by nanoindentation and MicroXCT™ suggest that the lesions have a highly demineralized region that extended approximately 80 μm followed by a gradual rise to normal dentin values at a depth of 140–150 μm.

### 3.1 Dentin shrinkage as an indicator of remineralization

**Figure 5:**
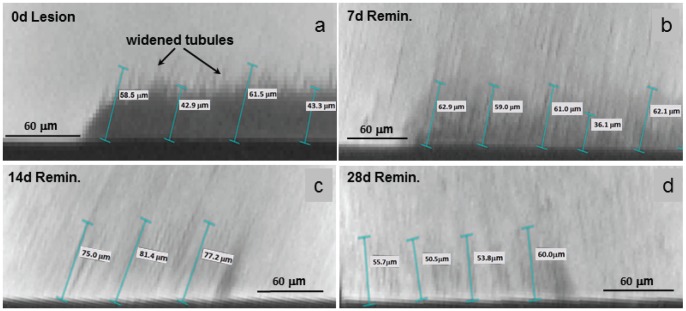
Typical 2d reconstructions showing x-ray attenuation with depth in the lesions at different remineralization times. a) control lesion (0 time) showing protected portion at the left, the severely demineralized region which gradually blends with normal dentin. Widened dentin tubules were apparent at the depth of the lesion. b) 7 day remineralization treatment seems more complete in the deeper part of the lesion. c) 14 day remineralization shows some unevenness in the remineralization, but much of the lesion appeared fully mineralized. d) 28 day remineralization was similar to 14 day specimens. Note there is no apparent shrinkage in any of the specimens as the imaging was done in fully hydrated tissue (see supplemental Figure 1). Dotted line shows approximate lesion depth, magnification bar = 100 µm. A movie at 10X, demonstrating widened tubules at the lesion site is also provided as supplemental information.

Table 1. shows the average depth of dentin shrinkage on dehydration for all treatment groups, as measured using light microscopy. The starting lesion depth underwent shrinkage of about 24 µm (Fig. 2). Shrinkage decreased markedly after 7 days and was only 1–2 µm at 14 or 28 days. This suggests that shrinkage is a reasonable indicator of mineral incorporation. The nanoindentation profiles presented in Figs. 3–5 were obtained from hydrated tissue and the distance within the lesion was corrected for any shrinkage for each specimen studied.

**Table 1:** Average shrinkage measurement at three time points relative to control

*Treatment Group*	*Average Shrinkage Measurement (µm)*
**Demineralized**	**24.3±1.0**
**7-D PILP**	**12.4±1.0**
**14-D PILP**	**1.7±0.4**
**28-D PILP**	**1.1±0.2**

### 3.2 Hydrated nanoindentation indicates functional remineralization of artificial caries

Figure 3 shows the reduced elastic modulus results for all groups studied. The most demineralized portion of the artificial lesions had values of about 0.2 GPa and spanned a depth of approximately 75–80 µm. Modulus values then increased gradually until normal values were obtained at a depth of about 140–150 µm. Control (undemineralized) values varied from about 15–20 GPa with an average of 17.9±0.1 GPa. With remineralization time the elastic modulus values increased throughout the lesion depth and displayed significant increases at each time period. Properties of the lesions appeared to increase from the depth of the lesion outward toward the surface as the gradient or sloped portion of the curve appeared to move toward the external surface and the low modulus flat region rose toward normal values reaching nearly 60% of normal values at the most demineralized surface. Hardness results for all groups studied had similar trends as those seen in the elastic modulus data (see supplementary data Fig. S2).

Table 2 shows the results and statistical comparisons from calculations of the differences in areas under the data curves for elastic modulus changes and hardness changes as a function of depth after 7, 14 and 28 days of remineralization. Improvements of 51% at 7 days, 74% at 14 days and 91% at 28 days were found for elastic modulus and improvements of 40% 46% and 77% for hardness at the same time periods were found as compared with the initial lesions.

**Table 2:** Analysis of the areas under the curves from the data of Figures 2, 3, and 7 with percent change from lesion (%).

*Measurement*	*Control*	*Lesion*	*7-Day PILP*	*14 Day PILP*	*28 Day PILP*
**E_R_ [GPa-µm]**	**3,220±100**	**1,170±140** *	**1,770±120** * **^+^ (51%)**	**2,040±50** * **^+^ (74%)**	**2,200±220** * **^+^ (91%)**
**H [GPa-µm]**	**130±6**	**46±5** *	**65±8** * **(40%)**	**68±3** * **(46%)**	**82±10** * **^+^ (77%)**
**Mineral Profile [HU- µm]**		**1,281,000±77,000**	**1,493,000±32,000^+^(17%)**	**1,622,000±20,000** * **(27%)**	**1,674,00±47,000^+^(31%)**

* Significantly different from control; + Significantly different from lesion, (p<0.05, n = 4, mean ± S.E.M).

To further determine the characteristics of the mechanical property recovery, the data were compared at each interval, namely, the changes in mean elastic modulus as a function of depth from 0–7 days, 7–14 days and 14–28 days. Figure 4 shows these incremental changes in E_R_ between remineralization intervals and demonstrated that within 7 days values increased predominantly in the sloped zone of the lesion (∼70–140 µm) as demonstrated in the green curve, while the 7–14 day period introduced more mineral in the outer portion of the lesion (blue curves) and the final period between 14 and 28 days contributed only minimally to the total regain of stiffness across the lesion. This suggests that functional remineralization occurred most rapidly in the sloped portion of the lesion and proceeded outward toward the external surface with time.

### 3.3 Mineral variations in demineralized and remineralized specimens

Typical MicroXCT™ images are shown for the artificial caries lesions (Fig. 5a) and the PILP remineralized lesions after 7, 14 and 28 days (Fig. 5b–d). Fig. 5a shows the highly demineralized region of the artificial caries lesion followed by a prominent increase in mineral with depth until the normal mineral level was reached. At this magnification the widened dentin tubules can be seen near the base of the lesion. At the left side of Fig. 5a the normal dentin that was protected from the demineralization treatment is evident. These images also show that the exposed surface was prepared nearly perpendicular to the tubule direction. MicroXCT™ images from the remineralized specimens revealed marked x-ray attenuation in the 7 day specimens (Fig. 5b), with further increases in the 14 (Fig. 5c) and 28 (Fig. 5d) day specimens with little difference apparent between the 14 and 28 day specimens.

**Figure 6:**
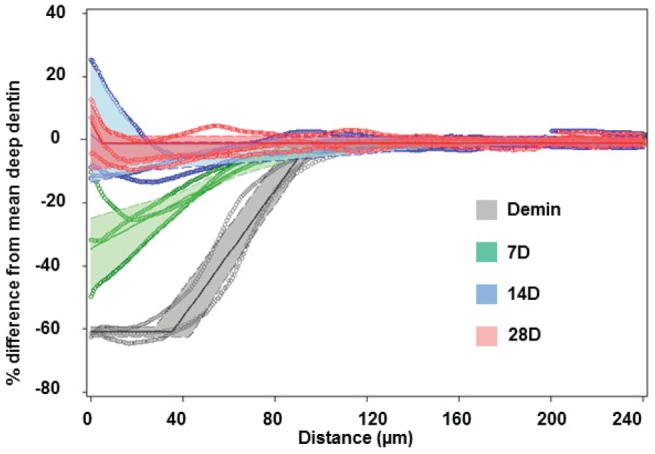
Mineral profiles derived from the MicroXCT™ virtual slices through the thickness of the lesion and remineralized specimens. The data is presented at baseline (grey) and at each remineralization period 7 (green), 14 (blue), 28 (pink) days is based on change-point models with 95% CLs.

Figure 6 shows the changes in mineral content at each time period of the PILP remineralization process based on analysis of the MicroXCT™ tomograms. The averaged mineral profile from MicroXCT™ indicated restoration of mineral starting from the deepest part of the lesion and providing nearly complete remineralization after 14 days with only minor additional gains in the following 14 days. It is interesting to note that the apparent depth of the lesions based on mineral profiles was about 110–120 µm, compared to the 140 µm that were apparent from the mechanical properties measurements (see Fig. 2). The major difference seems to be in the mineral content associated with the flat portion of the artificial lesion that is only about 40 µm, compared to about 70 µm in the flat portion as measured by nanoindentation. The sloped region in both cases is approximately 70 µm.

Figure 6 shows the mineral profiles derived from the MicroXCT™ virtual slices through the thickness of the lesion and remineralized specimens. The data is presented at baseline and at each remineralization period (7, 14, or 28 days) based on change- point models with 95% CLs. Similar trends in increased mineral level occurred at earlier time periods that corresponded to the increases in mechanical property data with the sloped region of the lesion gaining mineral most rapidly. However, there were two major differences. The initial lesion appeared to have a shallower flat external zone so that the total depth of the lesion appeared to be approximately 120 µm instead of 140 µm as seen in the mechanical profiles. 120 µm was the original target depth of the lesion as determined from prior kinetic studies of the demineralization buffer as determined from polarized light microscopy and the mineral profile from MicroXCT™ corresponded to the PLM data. In addition, unlike the mechanical data, there was full recovery of the mineral level throughout the lesion after 14 days and hypermineralization of the external region was apparent at both 14 and 28 days of remineralization (Fig. 6).

### 3.4 SEM/EDS and TEM/SAED characterization

**Figure 7:**
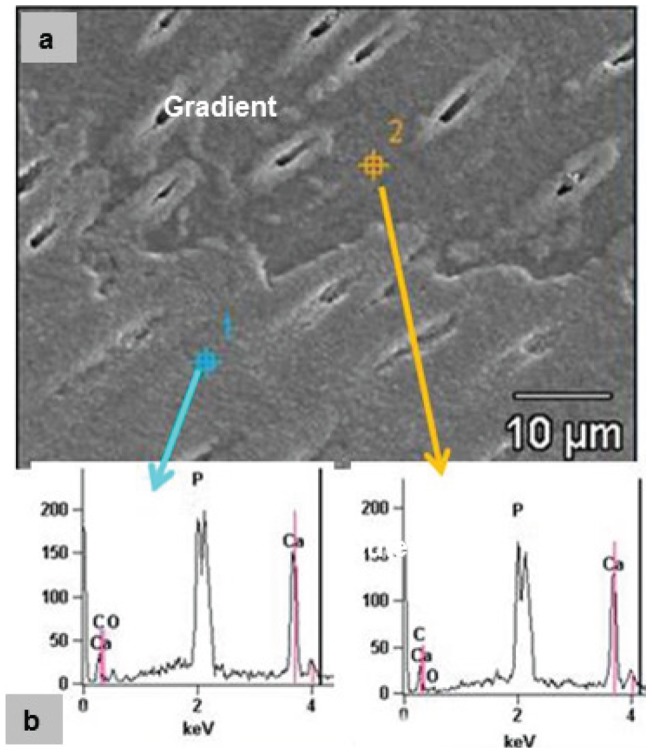
a) SEM showing marked structural differences in the outer (bottom) and gradient zones of remineralization. b) EDS analysis showed little difference at 14 days. Figs. 7a and 7b show outer zone mineral which seemed to cover the region and fill most of the tubule lumens. In the gradient zone the mineral formed a lip around each tubule lumen.

Scanning electron microscopy showed two distinct zones of remineralization as shown in Figure 7. In the SEM image (Fig. 7a), a wavy demarcation was clearly evident between the outer more demineralized portion (bottom) and the gradient portion of the remineralized lesion. However, both portions were significantly different than normal dentin or the initial lesion (not shown) and seemed to have pronounced mineralization within and around the periphery of each dentin tubule in the outer zone, and around the periphery of the tubules in the gradient zone. Energy dispersive analysis showed Ca and P present with no detected difference between these two remineralized zones and apparently normal dentin.

**Figure 8:**
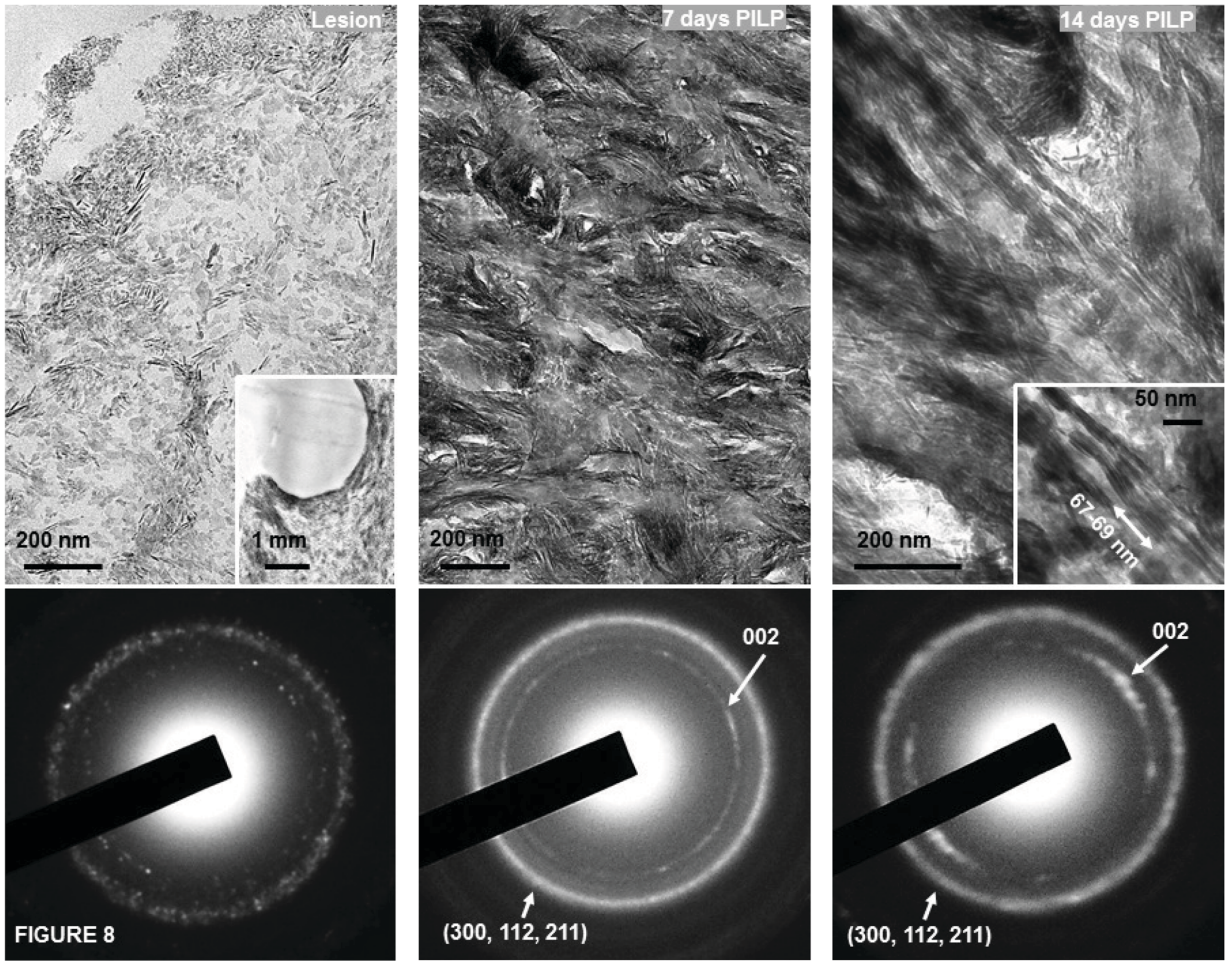
TEM of unstained lesion, 7 and 14 day specimens with corresponding SAED patterns showing intrafibrillar mineral within collagen fibrils after remineralization. Diffraction was consistent with apatite with its long axis along the axis of the collagen fibrils. Inset of lesion shows lower magnification of the specimen appearance and the inset at 14 days shows intrafibrillar crystallites at higher magnification.

TEM images of the initial lesion and remineralized specimens after 7 and 14 days are shown in Fig. 8 with associated electron diffraction patterns. The collagenous matrix of artificial caries lesion sites was severely demineralized but contained some residual minerals. Mineral was apparent in the fibrils after 7 days. After 14 days of PILP remineralization, collagen fibrils were filled completely with organized plate-like apatite crystals and showed characteristic 67-nm type-I collagen D-bands. The crystallinity and alignment of intrafibrillar minerals increased with growth time. The collagen fibrils in outer portion of the lesions were not fully mineralized even after 14 days, although total mineral content was normal as shown by MicroXCT™ (Fig. 6), which may account for the weaker mechanical properties.

## Discussion

This work determined that substantial restoration of the mechanical properties of hydrated carious dentin tissue occurred with the PILP process, thus providing functional remineralization [1], [2] in artificial lesions. Previously it has been observed that during demineralization the mineral is removed much more slowly from within the collagen fibrils than from the extrafibrillar compartment [28]. Mineral remaining within the collagen fibrils could act as nuclei for regrowth of the mineral and restoration of dentin properties [2].

Restoration of properties was evaluated using nanoindentations performed on the hydrated tissue, since dehydration alters the mechanical response of the collagenous matrix and provides little information about the functionality of dentin [2]. When carious or partially demineralized dentin is measured dry, the demineralized portion collapses and its measurement gives erroneously high values. Those values correlate linearly with total mineral content, but do not reflect the actual loss of stiffness and hardness due to missing mineral and lack of mineral coupling to the organic matrix. Thus impaired functionality of the tissue becomes evident when properties are measured under hydrated conditions, the natural state of dentin [1], [2], [29].

The changes in E_R_ of hydrated artificial caries specimens prepared using an acetate buffer (pH 5) that produces a sloped mineral profile similar to natural lesions were evaluated, although our artificial lesions had a larger and more pronounced outer zone of very low mechanical properties as compared to the natural caries previously studied [19], [24]. Artificial lesions offer significant advantages over natural lesions, since they are reproducible, and control lesions can be evaluated as well as the remineralized lesions. 140 μm lesions were used that provide sufficient depth to allow evaluation of remineralization changes at various time points.

When remineralized using the polymer-induced liquid-precursor (PILP) process the artificial lesions gradually recovered their mineral content and also recovered substantial mechanical properties that varied with location in the lesion, and included full recovery in the deeper portion and less recovery in the originally more demineralized portion. (Fig. 3 and supplementary Fig. S1). Property gains initially were most rapid in the gradient portion of the lesion and increased towards the surface with increased mineralization time between 7–14 days with smaller changes thereafter (Fig. 3). When shallower artificial lesions were remineralized without the polyaspartic acid additive, only minor mechanical properties improvement were observed at the surface, and often areas of mineral precipitation were apparent [30]. When 140 µm lesions were treated in the same way for 14 days only minor improvement was seen in the sloped portion of the specimens. In addition, SEM and EDS analysis showed clusters of HA crystals formed on the surface of the matrix when polyaspartic acid was not included. In contrast, no such precipitates formed on the matrix when the PILP process was used [31]. This strongly suggests that new mineral was deposited within the intrafibrillar and extrafibrillar portions of the matrix.

The averaged mineral profiles from MicroXCT™ indicated restoration of mineral starting from the deepest part of the lesion and providing nearly complete remineralization after 14 days with only minor additional gains in the following 14 days (Fig. 6). When the lesions' nanomechanical properties were analyzed in cross-sections, a gradient was observed in E_R_ and H, with properties decreasing from the normal tissue values along a slope towards the surface of the lesion similar to the mineral profile of the initial lesions. PILP remineralization led to recovery of the properties fairly rapidly within the first 7 days in a 70 µm zone close to the base or inner portion of the lesion, while stiffness increased in the rest of the lesion at a slower rate and reached only 50%–60% of normal tissue values with 14 or 28 days of remineralization. SEM showed distinct differences in the microstructure of the remineralized lesions (Fig. 7a) with tubules nearly filled and covered in the outer zone, and the periphery of the tubules showing a lip of mineral. Although EDS failed to show a difference in calcium or phosphate content between the two regions, both the remineralized outer and gradient zones were structurally distinct from normal dentin or the artificial caries lesion. Ultrastructural investigation by TEM (Fig. 7b) showed that the collagenous matrix at the acid-etched lesion sites was severely demineralized but contained some residual minerals; only after 14 days of PILP remineralization, collagen fibrils filled completely with organized plate-like apatite crystals and showed characteristic 67-nm type-I collagen D-bands. The crystallinity and alignment of intrafibrillar minerals increased with growth time. The collagen fibrils in outer portions of the lesions were not fully mineralized even after 14 days, although total mineral content was normal by Micro XCT, which may account for the weaker mechanical properties. This suggests that treatment length and lesion depth may affect the potential of dentin to be functionally remineralized.

Two zones existed within the artificial lesions that may interact differently with remineralization solutions. An inner zone at the bottom of the lesion is more readily remineralizable and fully recovers its properties at a rapid rate, while an outer zone requires additional time to improve in its mechanical response. Although mineral levels returned to normal in this outer zone, the properties did not fully recover at the longest time evaluated. Thus the mineral in this outer zone may not have been sufficiently incorporated into the collagen fibrils to provide complete recovery. This result might also reflect damage to the remaining collagen matrix in this outer zone that was severely demineralized. Several possibilities exist that we are currently investigating. Firstly, it has been established that dentin contains inactive matrix metaloproteinases [32]–[34]. and cysteine cathespins [35] that may be activated as mineral is lost due to demineralization at pH 5. Thus some of the collagen fibrils may be irreversibly damaged and therefore could not be completely functionally remineralized. In the inner zone the demineralization is much less severe and the remaining mineral prevents the protease activation. The use of protease inhibitors may alleviate this problem [36]. A second possibility is that removal of nearly all mineral in this zone allows for matrix expansion as water replaces mineral and the original organization is not fully recovered during demineralization. This idea is supported by TEM images showing a less dense matrix after remineralization of the outer zone (data not shown). A further possibility is that the intrafibrillar to extrafibrillar mineral ratios or crystallite sizes are not fully recovered. Each of these possibilities needs further investigation. Thus additional studies are warranted to identify structural differences in these two zones. Each zone may require a different strategy for functional remineralization. The highly demineralized superficial zone and partially demineralized zone represented by the regions of increased slope in the artificial lesion profiles are likely to correspond to the intensely stained zone, and less stained inner zones, respectively, that are identified by caries detector in clinical caries [19], [24].

There was a significant discrepancy between mineral profiles and the mechanical properties profiles at each time point. The lesions measured by mineral profile appeared shallower, particularly in the outer zone as compared to mechanical properties profiles using E_R_ and H. This suggests that the main difference was in this outer zone and depended on the difference in detection of the two methods. It is likely that the lesions appear deeper by nanoindentation because a critical mineral level must be reached before the indentation force can be resisted. In addition it should be noted that the two methods utilized on the same specimens do not measure exactly the same locations or tissue volumes as illustrated in Fig. 1c and d.

### Conclusions

Within the limitations of the present experiments, the following could be concluded: functional remineralization of partially demineralized human dentin occurred with recovery of mechanical properties, with progressive intra- and extra-fibrillar mineralization initiated in the depth of the lesion. The degree of remineralization increased with time over the 4 week treatment period. Approximately half of the lesion depth (in a total depth of 140 µm) recovered to normal levels of E_R_, while the outer portion recovered about 50% of its mechanical properties during the remineralization period, even though normal mineral recovery was achieved. Results suggest that functional remineralization through shallow lesions is possible within 2–4 weeks, demonstrating the clinical translation potential of the proposed mechanism.

## Supporting Information

Figure S1 a). Specimen containing lesion in a micropipette tip filled with water. b). Specimen within the micropipette shown at a higher magnification. Inset illustrates lesion with widened tubules due to demineralization of peritubular dentin that form funnel shapes that transit into normal tubules with depth (see Movie S1).
(TIF)

Figure S2 Hardness changes as a function of treatment time from 0–28 days shows the changes in hardness (H) at each of the remineralization periods as compared to the starting lesion (grey). Trends were similar to those seen in the modulus values. Between 0 and 7days (green) most of the changes occurred in the depth or inner part of the lesion. The following 7 day period (blue) showed increases mainly in the outer zone and the final 7 day period (red) contributed only minor additional increases in the mechanical properties. This suggests that initially functional remineralization resulted in mineral being deposited in the inner portions of the lesion and this deposition gradually moved outward toward the surface.
(TIF)

Movie S1 Movie illustrates progressive virtual sections from the external surface to the deeper portions of the demineralized specimen. Widened dentin tubules due to demineralization of the peritubular dentin forms funnel shapes that transit into normal dentin with depth.
(MPG)
