# Correction: Functional Remineralization of Dentin Lesions Using Polymer-Induced Liquid-Precursor Process

**DOI:** 10.1371/annotation/b51e432d-e5b8-4579-927d-37a0a7a4323d

**Published:** 2013-05-03

**Authors:** Anora K. Burwell, Taili Thula-Mata, Laurie B. Gower, Stefan Habeliz, Michael Kurylo, Sunita P. Ho, Yung-Ching Chien, Jing Cheng, Nancy F. Cheng, Stuart A. Gansky, Sally J. Marshall, Grayson W. Marshall

There was an error in the same of the fourth author.

The correct name is: Stefan Habelitz

The correct citation is: Burwell AK, Thula-Mata T, Gower LB, Habelitz S, Kurylo M, et al. (2012) Functional Remineralization of Dentin Lesions Using Polymer-Induced Liquid-Precursor Process. PLoS ONE 7(6): e38852. doi:10.1371/journal.pone.0038852 

