# Supplementary figures and images for: Functional Remineralization of Dentin Lesions Using Polymer-Induced Liquid-Precursor Process
Source: PLoS One. 2012 Jun 13;7(6):e38852. doi: 10.1371/journal.pone.0038852 (PMC3374775; doi:10.1371/journal.pone.0038852)

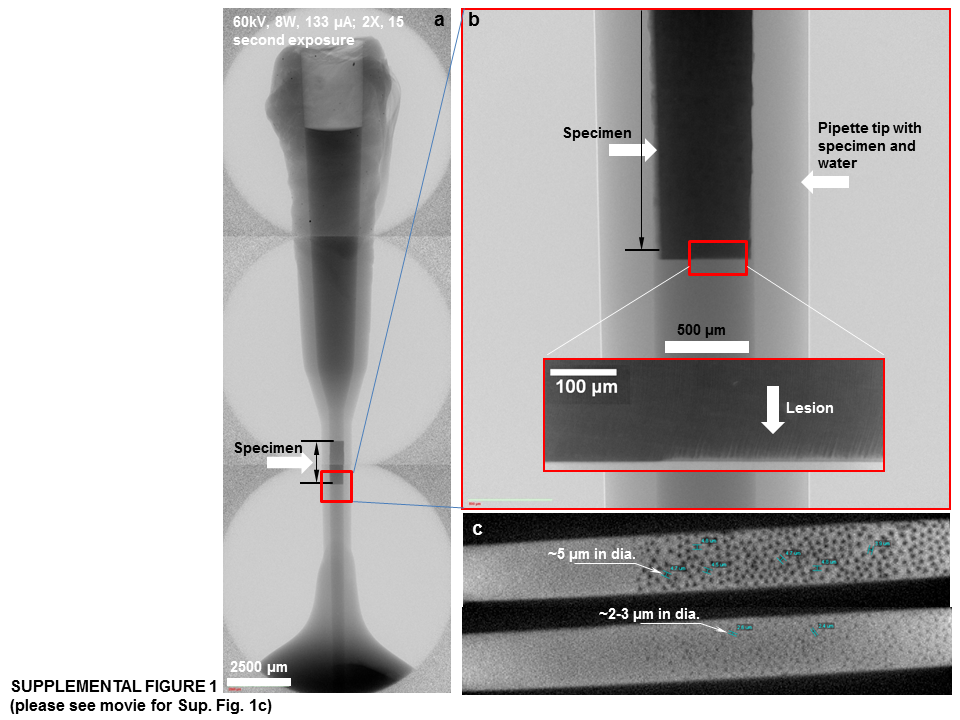

Supplement: Figure S1 — a). Specimen containing lesion in a micropipette tip filled with water. b). Specimen within the micropipette shown at a higher magnification. Inset illustrates lesion with widened tubules due to demineralization of peritubular dentin that form funnel shapes that transit into normal tubules with depth (see Movie S1). (TIF) [file pone.0038852.s001.tif]

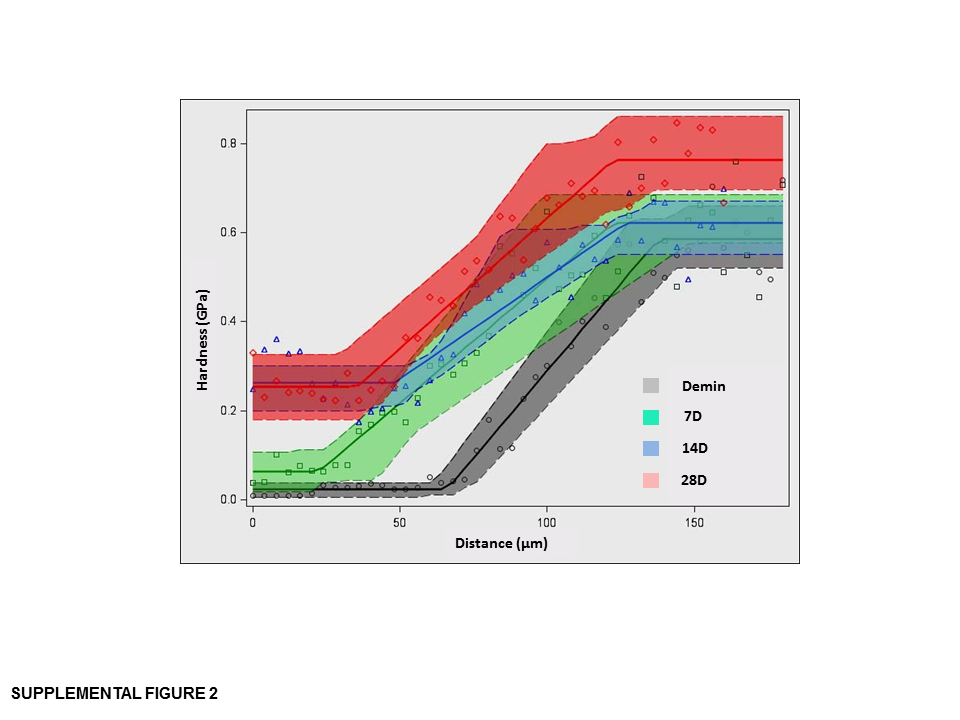

Supplement: Figure S2 — Hardness changes as a function of treatment time from 0–28 days shows the changes in hardness (H) at each of the remineralization periods as compared to the starting lesion (grey). Trends were similar to those seen in the modulus values. Between 0 and 7days (green) most of the changes occurred in the depth or inner part of the lesion. The following 7 day period (blue) showed increases mainly in the outer zone and the final 7 day period (red) contributed only minor additional increases in the mechanical properties. This suggests that initially functional remineralization resulted in mineral being deposited in the inner portions of the lesion and this deposition gradually moved outward toward the surface. (TIF) [file pone.0038852.s002.tif]
